# Nature-inspired wax-coated jute bags for reducing post-harvest storage losses

**DOI:** 10.1038/s41598-021-93247-z

**Published:** 2021-07-28

**Authors:** Kennedy Odokonyero, Adair Gallo, Himanshu Mishra

**Affiliations:** Interfacial Lab, Water Desalination and Reuse Center, Division of Biological and Environmental Science and Engineering, King Abdullah University of Science and Technology (KAUST), Thuwal, 23955-6900 Saudi Arabia

**Keywords:** Bioinspired materials, Nanoscale materials, Ecophysiology, Environmental sciences, Tropical ecology, Chemical modification

## Abstract

Post-harvest storage of grains is crucial for food and feed reserves and facilitating seeds for planting. Ironically, post-harvest losses continue to be a major food security threat in the developing world, especially where jute bags are utilized. While jute fabrics flaunt mechanical strength and eco-friendliness, their water-loving nature has proven to be their Achilles heel. Increased relative humidity and/or precipitation wets jute, thereby elevating the moisture content of stored seeds and causing fungal growth. This reduces seed longevity, viability, and nutritional value. To address this crucial weakness of jute bags, we followed a nature-inspired approach to modify their surface microtexture and chemical make-up via alkali and wax treatments, respectively. The resulting wax-coated jute bags (WCJBs) exhibited significant water-repellency to simulated rainfall and airborne moisture compared to control jute bags (CJBs). A 2 months-long seed storage experiment with wheat (*Triticum aestivum*) grains exposed to 55%, 75%, and 98% relative humidity environments revealed that the grains stored in the WCJBs exhibited 7.5–4% lesser (absolute) moisture content than those in the CJBs. Furthermore, WCJBs-stored grains exhibited a 35–12% enhancement in their germination efficacy over the controls. This nature-inspired engineering solution could contribute towards reducing post-harvest losses in the developing world, where jute bags are extensively utilized for grain storage.

## Introduction

In 2020, the Nobel Peace Prize was awarded to the World Food Programme, underscoring that some fractions of humanity are still deprived of food security^1^. According to the United Nation’s Food and Agricultural Organization, developing sustainable agricultural practices to feed ~ 10^10^ humans in 2050 will be a mega-challenge of our lifetime; it is anticipated that annual food production worldwide must increase by approximately 70%^2–5^. At present, grain crops such as cereals, oilseeds, and pulses, which form the basis of global food security^6–8^, are produced at the scale of ~ 2 B tons/year^7,9^. By 2050, this is projected to increase by > 33%^3,10^. However, increasing crop yields beyond current levels is likely to be difficult due to climate change, groundwater depletion and pollution, and limited energy resources^11–14^. Food security in the emerging scenario will mandate a highly efficient supply chain from farm to consumer^8^.

Currently, post-harvest losses (PHLs), which entail quantitative and qualitative losses from harvest through to storage, processing, marketing, and consumption, claim 25–33% of the entire grains produced globally^6,15–18^. In developed countries, grain losses during storage can be as low as 1–2% because of the application of modern technologies^9,19^. In developing countries, contrastingly, up to 60% of cereals may be lost during storage due to poor storage infrastructure^16–18,20^, slower adoption of modern technologies^21^, and fragmented information sharing^19^. PHLs can be in the form of spoilage, seed viability loss, grain depletion, and nutritional loss^22^ resulting from biotic (e.g., insects, rodents, and fungi) or abiotic (e.g., temperature and air-moisture/humidity) factors^9^. Fungal infections account for 25–40% of PHLs^23,24^, and mycotoxin contamination can pose a significant health risk^25^.

Airborne water vapor (mass/volume)—commonly expressed as relative humidity (RH: the ratio of the partial pressure of water vapor to the saturation vapor pressure at a given temperature)—crucially influences the equilibrium seed moisture content (SMC); the specifics of water uptake vary with seed type and temperature^26,27^. Elevated SMC promotes the growth of bacteria, fungi, and insects and vice versa. For example, storage at 30 °C below 65% RH corresponds to an equilibrium SMC of 12–14% in cereals, which prevents fungal infestation^15,27,28^; on the other hand, storage at < 35% RH (or < 8% SMC)^27–29^ arrests the growth of insects on top of fungi. In contrast, at RH > 65%, as SMC exceeds 14%, storage bacteria, fungi, and insects start respiring and increasing temperature and deteriorating grains^15,30,31^. For example, the germination capacity of soybean (*Glycine max*), sorghum (*Sorghum bicolor*), red clover (*Trifolium pratense*), and Timothy grass (*Phleum pratense*) seeds reduced substantially during 6–12 months of storage at 30 °C and 75% RH^32^. In this context, Harrington’s thumb rule states that for every 1% increase in SMC, shelf life decreases by 50%^30,31,33–35^; this exponential relationship underscores the role of storage bags in acting as a diffusion barrier for water vapor.

To keep SMC under check, the concept of “dry chain”—akin to “cold chain” for perishable products—has been put forth, wherein grains are dried upon harvest followed by the use of moisture-proof packaging until consumption^28,36^. Furthermore, to combat humid environments, researchers have developed moisture- and oxygen-proof hermetic storage systems such as GrainPro Superbags™ and Purdue Improved Crop Storage (PICS) bags^28,36–40^. However, these polypropylene bags are vulnerable to puncturing from sharp objects and rodents; cost and disposal also prevent widespread adoption. Therefore, low-cost solutions for preventing PHLs are still needed.

In the Indian subcontinent and sub-Saharan Africa, farmers exploit jute bags for grain storage because of their low cost, mechanical durability, and traditional usage over millennia^9,21,41^. These bags are stacked in traditional storage structures or outdoor stockpiles covered with tarpaulin^9,21^. Jute is water-loving (hydrophilic) in nature; and it also absorbs water due to its constituents—cellulose (~ 60%), hemicellulose (~ 20%), lignin (~ 10%), pectin, and water-soluble substances^42–45^. Stored grains are often exposed to high humidity, especially in the event of precipitation or increased RH, which wets the bags and elevates SMC, accelerates seed deterioration, and reduces germination efficacy^28,32,46^. In India, for example, nearly 60–70% of all the grains produced are stored in conventional structures of which ~ 35% of rice and wheat grains get damaged by PHLs^47^.

To address this weakness of jute bags, we took inspiration from nature—the various plants and animals that have evolved water-repellency by combining hydrophobic waxy coatings with micro/nanoscale surface roughness. Examples include lotus leaves (*Nelumbo nucifera*)^48^, bodies of sea-skaters (*Halobates germanus*)^49^, cuticles of springtails (*Collembola*)^50^, as well as certain desert plants^51^ and insects^52^, among others^53^. In this work, jute fabrics were treated with alkalis to induce surface roughness, followed by coating with paraffin wax to engender water repellency. The seed storage efficacy of these wax-coated jute bags (WCJBs) was then compared with that of control jute bags (CJBs, i.e., untreated jute bags) under specific relative humidity conditions over a 2-months-long storage duration. This was followed by a germination study in which the viability of seeds stored in WCJBs and CJBs was comparatively assessed.

## Results

### Water repellency

**Figure 1:**
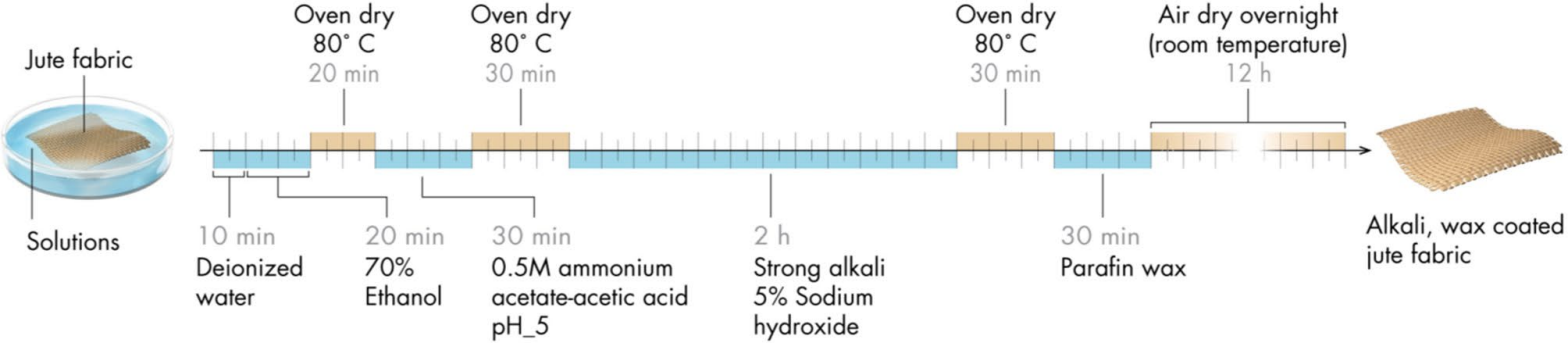
Schematic of the jute surface treatment process (image credits: Xavier Pita, KAUST).

Our study demonstrates a simple surface treatment protocol to produce hydrophobic WCJBs through chemical treatment with alkali, followed by surface coating using paraffin wax that is inexpensive and readily available (Fig. 1).

**Figure 2:**
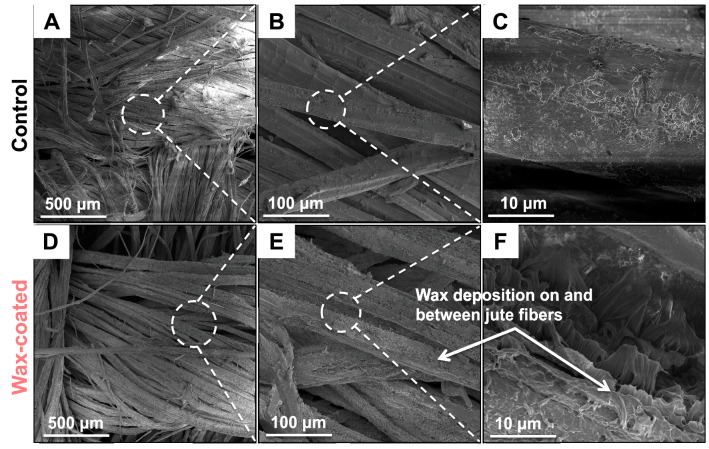
Representative scanning electron micrographs of jute fiber surface morphologies of control jute bags (CJBs) and wax-coated jute bags (WCJBs). Fibers of CJBs (**A**–**C**) show lower surface roughness in comparison to WCJB fibers (**D**–**F**). Wax deposits are also indicated with arrows.

After wax coating, the characterization of jute surface morphologies using SEM revealed a smooth fiber surface in the CJBs while WCJBs appeared rougher (Fig. 2). Gaps between WCJB fibers (Fig. 2D–F) reduced in comparison to those between CJB fibers (Fig. 2A–C) because wax coating tends to bind adjacent fibers. In addition, following the alkali treatment and wax coating, fibers of WCJBs appeared swollen (Fig. 2D) than of CJBs (Fig. 2A).

**Figure 3:**
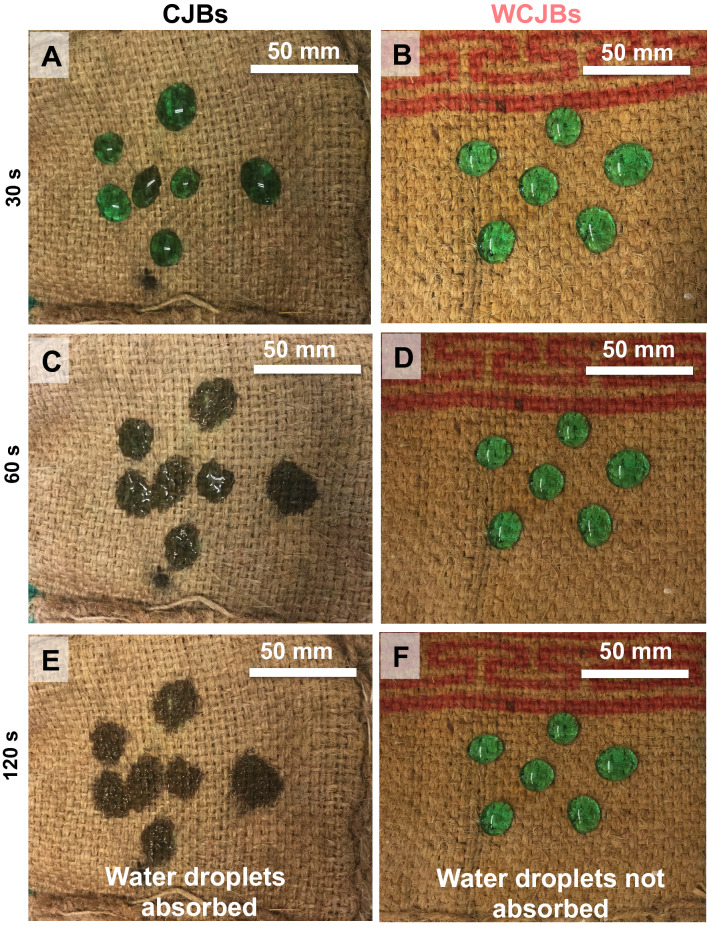
Representative photographs showing time-dependent wetting of control jute bags (CJBs) and wax-coated jute bags (WCJBs). At 30 s, water droplets began to seep into the CJBs, whereas droplets could not penetrate into the WCJBs (**A**,**B**). Within 1 min, CJBs showed significant wetting; in contrast, water droplets did not spread on the WCJBs (**C**,**D**). From 2 min onwards, water droplets were completely absorbed by the CJBs, whereas the droplets on WCJBs could not penetrate even after > 3 h (**E**,**F**). (Note: we added dye to color the water in order to serve as a visual aid; experiments without dye yielded identical results).

When water droplets of ~ 8 μL were placed onto CJBs, they gradually penetrated into the fibers in ≤ 2 min (Fig. 3A,C,E), while WCJBs significantly repelled water droplets as demonstrated by water droplets failing to penetrate even after > 3 h during which they evaporated (Fig. 3B,D,F). Furthermore, when CJBs and WCJBs were exposed to simulated rainfall, the CJBs got wet by impacting water droplets that they accumulated (Movie 1). In contrast, water droplets simply rolled-off the WCJBs on impact, thereby preventing water accumulation (Movie 2).

### Seed storage experiments

**Figure 4:**
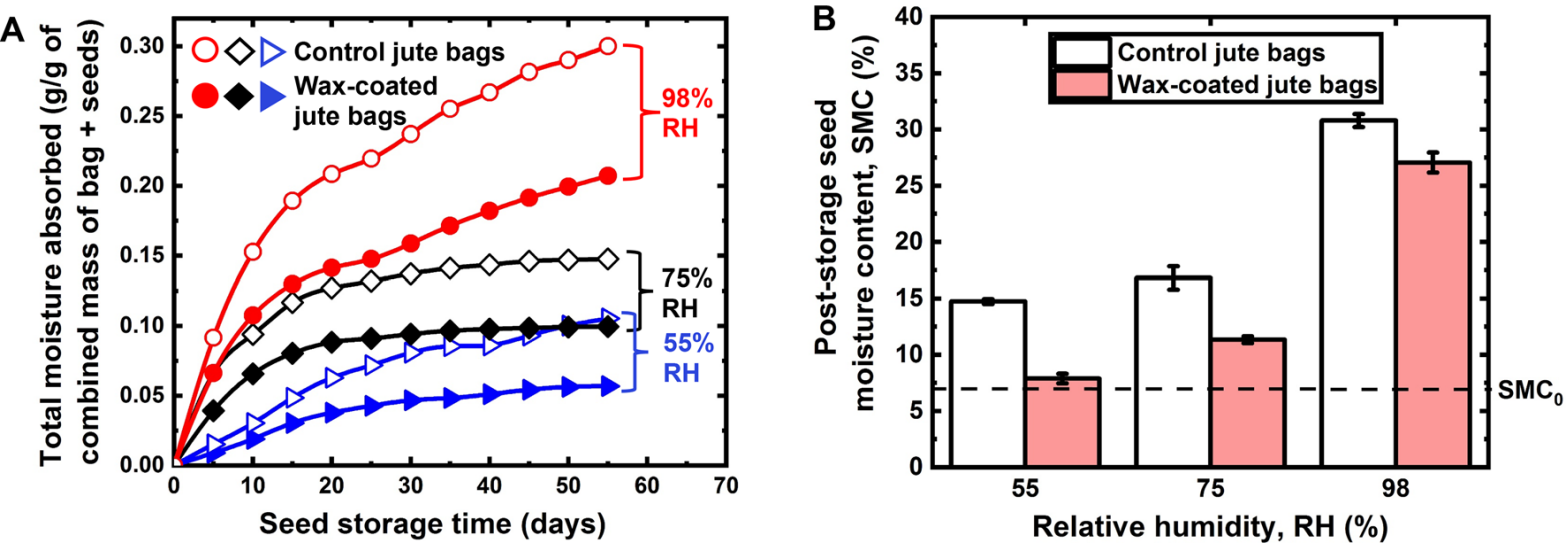
Moisture absorption through jute bags and the moisture content of wheat grains after 2 months of storage. (**A**) Total moisture absorbed through control jute bags (CJBs: unfilled shapes) and wax-coated jute bags (WCJBs: filled shapes) over the seed-storage period. Total moisture absorbed was taken as the sum of moisture uptake by jute bags and stored grains; the combined mean mass of the storage bag plus seed was ~ 170 g/bag. (**B**) Post-storage seed moisture content (SMC) of stored wheat grains at different relative humidity (RH) conditions. Horizontally-dotted line (SMC_0_) represents initial SMC (7.1%) before storage; error bars represent the standard errors (± SE) of means from three replicates.

After comparing the wetting behaviors, we hypothesized that WCJBs should have superior potential for protecting stored grains from humidity than CJBs. Wheat (*T. aestivum*) grains were stored for 2 months in WCJBs and CJBs (controls) at 21.6 ± 0.5 °C under three different relative humidity conditions: 55%, 75%, and 98%. The SMC of the wheat prior to storage was 7.1%. During the study, we quantified changes in the mass of the storage bags containing grains every 3 days and also looked for signs of moisture accumulation on the bags, dampness, and fungal (mold) growth (Figs. 4, Fig. S1).

### Seed moisture content

For the 55% RH treatment, the total moisture absorbed by the WCJB + grain system was 46% lower than that of the CJB + grain system (Fig. 4A); the SMC of the grains stored in the WCJBs increased from 7.1 to 7.5%, whereas the SMC of the grains in the CJBs increased from 7.1 to 14.9% (Fig. 4B). Crucially, CJBs absorbed 12-times more moisture than that absorbed by WCJBs; thus, they acted as reservoirs for moisture influx into stored grains. Next, for the 75% RH treatment, the total moisture absorbed by the WCJB + grain system was 32% lower than the controls; the SMC of the grains stored in the WCJBs increased from 7.1 to 11%, whereas the SMC of the grains in the CJBs increased to 17%. Lastly, for the 98% RH treatment, the total moisture absorbed by the WCJB + grain system was 45% lower than the controls; the SMC of the grains stored in the WCJBs increased to 26%, whereas the controls increased to 32%. These findings demonstrate that WCJBs provide superior protection to stored grains than CJBs at $\le$ 75% RH.

### Fungal infestation

**Figure 5:**
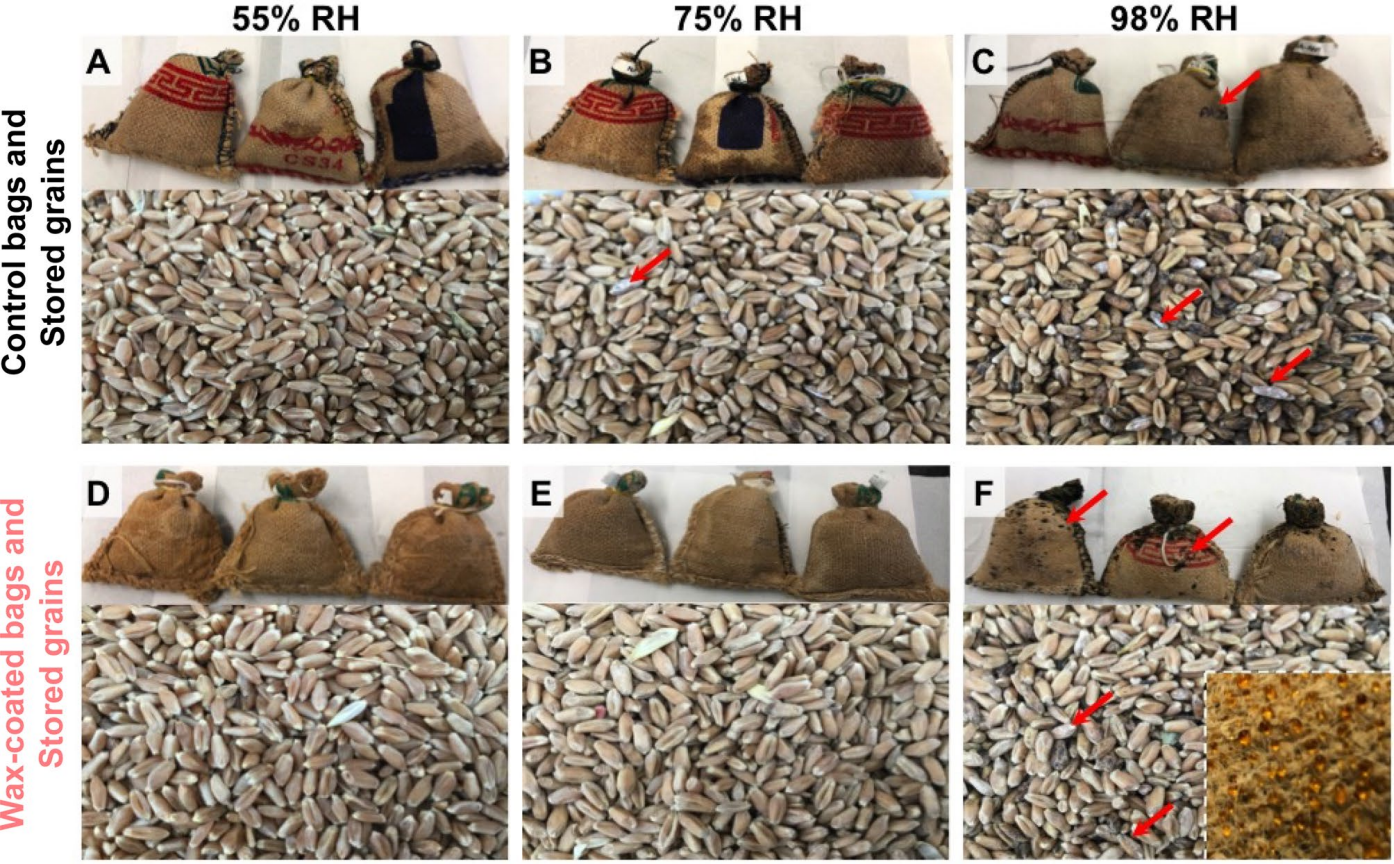
Photographs of wax-coated jute bags (WCJBs) and control jute bags (CJBs) with stored wheat grains after 8 weeks of storage under different relative humidity (RH) conditions. CJBs with wheat grains at 55% RH (**A**), 75% RH (**B**), and 98% RH (**C**); WCJBs and wheat grains stored at 55% RH (**D**), 75 % RH (**E**), and 98% RH (**F**). Red arrows indicate fungal (mold) growth. At 55% RH, neither WCJBs nor CJBs suffered from dampness or fungal growth, although seed moisture content increased from 7.1% to 7.5% in WCJBs and 14.9% in CJBs. WCJBs also performed well at 75% RH; however, grains stored in CJBs at 75% RH showed signs of fungal growth. At 98% RH, both WCJBs and CJBs and their stored grains exhibited dampness and fungal growth (dark grey patches). The inset photograph in (**F**) shows the accumulation of moisture on WCJBs in the third week of storage.

Due to their water repellency, WCJBs absorbed significantly lesser water than CJBs from the air during storage under different RH conditions. For the 55% RH treatment, we did not observe signs of fungal mold on bags or grains in WCJBs or CJBs (Fig. 5A,D). For the 75% RH treatment, the WCJBs and stored grains did not exhibit mold growth, whereas the CJBs and stored grains showed dampness and fungal growth towards the end of the study (Fig. 5B,E). Tiny droplets of condensed water appeared onto WCJBs towards the end of the study, as expected for hydrophobic surfaces but the droplets did not imbibe into the bag's surface^54^. For the 98% RH treatment, both WCJBs and CJBs and stored grains exhibited dampness and fungal growth (Fig. 5C,F). Condensed water droplets appeared on WCJBs after 2 weeks (Fig. 5F inset), and as they grew larger, many rolled off the surface. Fungal proliferation on the grains stored inside WCJBs was less prominent than that on the grains stored inside CJBs.

### Seed germination efficacy

To further explore the effects of storage on seed health after our 2 months-long study, we investigated seed germination efficacy using the following formula:

$$Seed~Germination~Efficacy,~SGE = \frac{{{\text{Number~of~germinated~seeds}}}}{{{\text{Total~number~of~seeds~sown}}}} \times 100$$

**Figure 6:**
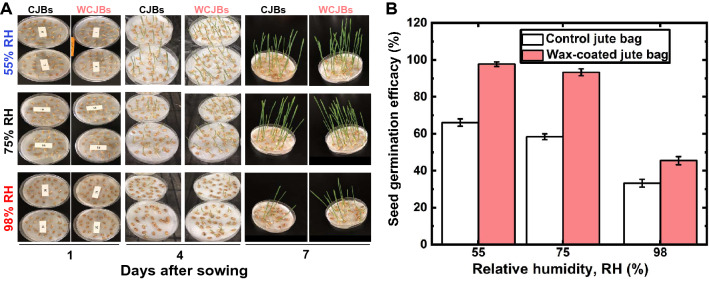
Seed germination on Petri dishes of wheat grains stored for 8 weeks in control jute bags (CJBs) and wax-coated jute bags (WCJBs) under different relative humidity (RH) conditions. (**A**) Seed germination patterns monitored for 7 days after sowing and (**B**) germination of seeds stored in control jute bags and WCJBs at 55%, 75%, and 98% RH. The percentage seed germination of wheat grains stored in WCJBs was higher than that of wheat grains stored in CJBs under all RHs. Error bars represent the standard error (± SE) of means from 90 seeds/treatment.

In a 1 week-long study of germination (Fig. 6A), we found that grains stored in WCJBs performed better than those stored in CJBs (*p* < 0.05). For 55%, 75%, and 98% RH conditions, WCJBs afforded 32%, 35%, and 12% higher germination efficacy, respectively (Fig. 6B).

## Discussion

Here we discuss the factors and mechanisms underlying the superior performance of WCJBs at safeguarding grains and seed germination efficacy. First, we discuss the effects of alkali and wax treatments on jute surface properties. Alkali treatment of cellulosic fibers, also known as mercerization, has been exploited to tune various fiber characteristics, such as elastic moduli, brittleness, surface roughness, and readiness for surface coating, of jute and cotton^44,55–58^. It dissolves hemicellulose and other fiber components causing swelling and constriction of pores in jute fabrics^44,59–61^. Researchers have demonstrated that mechanical properties such as tensile strength, flexural strength, tensile modulus, and flexural modulus are superior for 6% NaOH-treated jute fabric reinforced composites relative to the jute fabrics treated at higher alkali concentrations (> 6%)^62^. Therefore, we chose alkali concentration of 5% and treatment duration of 2 h to enhance surface roughness without significantly depleting mechanical strength^44,59,63^.

**Figure 7:**
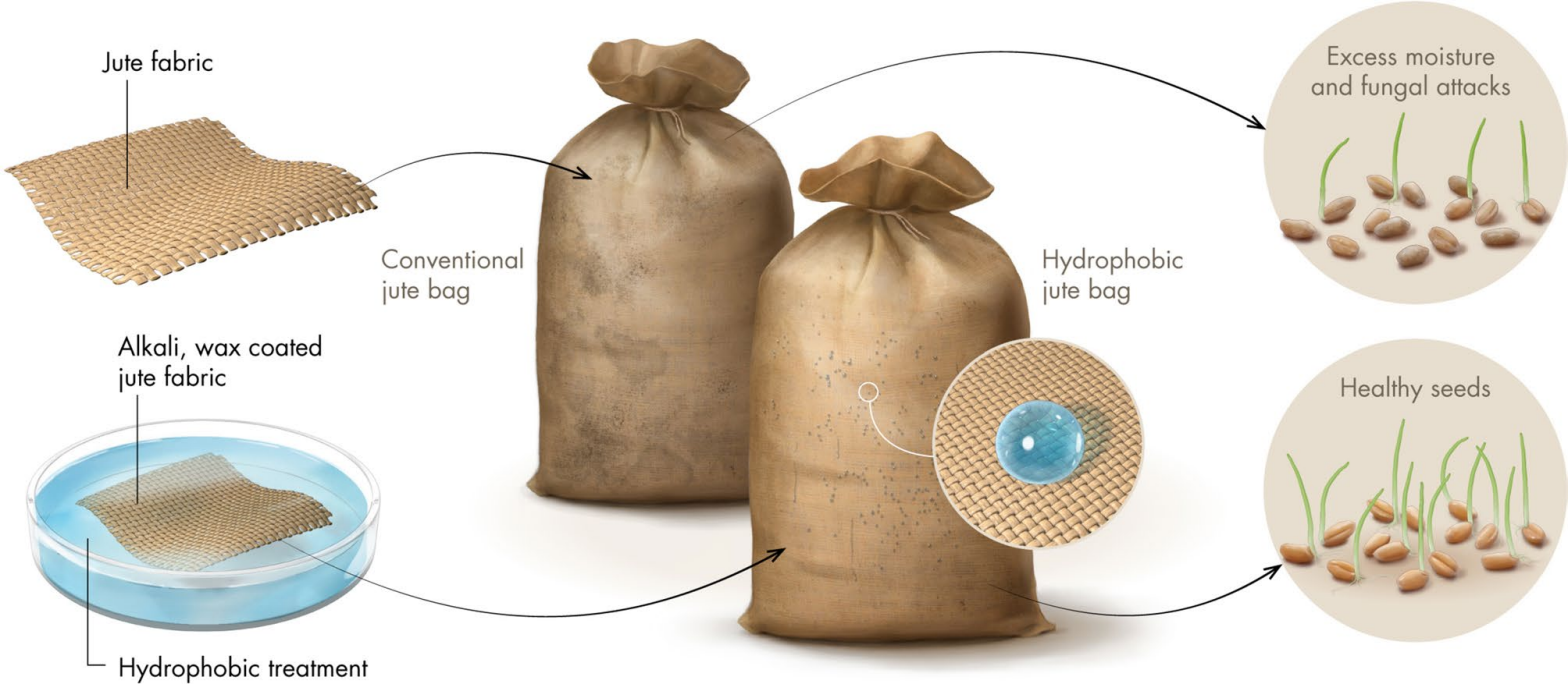
Schematic showing the surface treatments of jute bags with alkali and wax coating, the seed storage experiment, and the germination experiment for stored wheat grains. Control jute bags, CJBs; hydrophobic (wax-coated) jute bags, WCJBs. While CJBs accumulate water and get wet, WCJBs prevent water accumulation and cause water droplets to roll off their surface. Post-storage seeds from WCJBs are more likely to have higher germination than seeds from CJBs (image credits: Xavier Pita, KAUST).

As alkali-treated jute fabrics were coated with paraffin wax, the combination of surface roughness and the hydrophobicity of the wax produced robust water repellency^64^. The intrinsic hydrophobicity of paraffin wax is characterized by the apparent contact angles of water droplets on smooth wax-coated surfaces: ${\theta }_{o}\approx 105^\circ$ ^65^. When a rough surface is coated with a hydrophobic coating, the resulting water-repellency is enhanced by the entrapment of air inside the surface asperities, which also prevent the penetration of water into the microtexture^66^. While water droplets rapidly penetrated the CJBs, water droplets placed onto WCJBs did not penetrate into the fibers due to the entrapment of air in the microtexture. Apparent contact angles of water drops were ${\theta }_{\mathrm{r}}\approx {130}^{^\circ }$ and they remained pinned as they evaporated (Fig. 3B,D,F). These air-trapped wetting states are described by the Cassie–Baxter model, which connects the macroscopic apparent contact angles on the rough surface, ${\theta }_{\mathrm{r}}$, with ${\theta }_{\mathrm{o}}$ and the area fractions of liquid–solid (${\phi }_{\mathrm{L}\mathrm{S}}$) and liquid–vapor (${\phi }_{\mathrm{L}\mathrm{V}}$) fractions underneath the drop as $\mathrm{cos}{\theta }_{\mathrm{r}}={\phi }_{\mathrm{L}\mathrm{S}}\times \mathrm{cos}{\theta }_{o}-{\phi }_{\mathrm{L}\mathrm{V}}$^67^. If the impacting droplets have momentum, they even bounce off the surface^68^. Therefore, WCJBs are not as vulnerable to precipitation as CJBs (Fig. 7).

Water condensing on CJBs surface is expected to form thick films due to jute’s hydrophilicity^54^. Thus, CJBs appeared damp in the storage experiments and acted as reservoirs for moisture for the seeds stored inside. In contrast, water condensed as discrete droplets on WCJBs due to their hydrophobicity. As these droplets grew larger over time or due to coalescence, many of them rolled off as their weight approached the magnitude of pinning forces, which reduced the effect on the SMC of stored grains.

We also noticed that the fibers of WCJBs were somewhat swollen in contrast to those of CJBs (Fig. 2). This likely led to partial pore clogging of the treated-jute layer and reduced the flux of water vapor through the pores^69^. Additionally, paraffin wax seeping through pores from both surfaces could have further constricted the pores. This hypothesis is consistent with previous findings, where the durability of jute geotextiles was studied in soil, and the authors noticed that impregnation of nonwoven jute samples with bitumen reduced jute pore sizes because of the formation of a superimposing bitumen binder layer on the surface of both sides of the jute fabric^70^.

Lastly, we note that the differences in the SMC of wheat grains stored in WCJBs and CJBs were quite large, but this did not translate into exponential trends in germination efficacy as expected by Harrington’s thumb rule. This was presumably due to the short duration of our storage experiment, i.e., fungal infestation did not severely deteriorate the stored seeds. Thus, experiments with longer storage duration are warranted to probe this further.

## Conclusion

Principles underlying water repellency in various animals and plants have been harnessed for developing technologies for separation and purification^64,71^, desalination^72–74^, cavitation mitigation^75^, coating-free entrapment of air underwater^50,76,77^, and producing super-water-repellent cotton^56^, among others^65^. However, their application in agricultural engineering and technology has remained limited^78,79^. This proof-of-concept study demonstrates that WCJBs can substantially extend the shelf life and germination efficacy of seeds stored under low RH conditions and facilitate increased resilience under higher RH conditions. We hope that these results will inspire multidisciplinary research towards identifying strengths and weaknesses of this approach. Many questions remain unanswered such as: How SMC and seed germination efficacy vary over longer experimental duration and with seed type?; How to dispose/recycle WCJBs?; Would wax coatings degrade during handling/transportation?; What is the simplest process for applying wax on jute fabrics?; How does the cost of WCJBs compare with alternatives, such as PICS? Life cycle analysis of wax-coated jute and its other water-proofing applications should also be explored. We hope that this nature-inspired approach can help us realize jute’s full potential for grain storage and beyond.

## Methods

### Materials and apparatus

Paraffin wax was obtained from common white candles manufactured by IKEA (Sweden); hexane and ethanol were purchased from VWR International S.A.S (Fontenay-Sous-Bois, France); ammonium acetate, glacial acetic acid, and calcium nitrate tetrahydrate (Ca(NO_3_)_2_·4H_2_O)) were purchased from Sigma Aldrich (St. Louis, MO, USA); sodium chloride, potassium sulfate, and sodium hydroxide were purchased from Fisher Scientific (New Jersey, USA). All chemicals were used as received from suppliers. Jute packaging sacks (*Indian Sela Basmati Rice*, 50 kg) were obtained from the *Al Jawahir* Company (Saudi Arabia). A Quanta 600 scanning electron microscope system was used for scanning electron microscopy. A Quorum Q150 TS sputter coater was used to coat jute bags (CJBs and WCJBs) with a 3 nm-thick platinum layer to avoid electrical charging during electron microscopy. A DSA100E contact angle goniometer (Kruss, Germany) was used to characterize the wettability of surfaces. Wheat grains (*Triticum aestivum* L.) were harvested from a field at King Abdulaziz University’s Agricultural Research Station in Hada Al-Sham (which extends between 39**° **30′ and 40**° **15′ East and 21**° **45′ and 22**° **10′ North, in Makkah, Saudi Arabia).

### Surface treatment of jute

Jute sacks (fabric thickness ~ 11.28 ± 0.06 mm) were cut into 12.6 cm × 10.5 cm pieces and hand-sewn to produce jute storage bags (483 ± 97 g/bag). First, the bags were cleaned by immersion in water (10 min) and then 70% ethanol (20 min), before being dried in a thermal convection oven at 80 °C (30 min). Cleaned bags were then immersed in 0.5-M ammonium acetate solution (adjusted to pH 5 using 1.0% glacial acetic acid) for 30 min. After further oven drying at 80 °C (30 min), the bags were immersed in 5% (m/v) NaOH solution (1.25 M and pH ~ 14) for 2 h. These alkali-treated bags were washed with deionized water and again oven-dried at 80 °C (30 min). Subsequently, the bags were immersed in solutions of paraffin wax dissolved in hexane (6 g/L), during which they were turned upside-down several times to facilitate uniform coating. After 30 min of this treatment, the bags had absorbed ~ 100 mL of the wax solution and were left to dry overnight in a solvent hood at 21.6 °C ± 0.5 °C.

### Wettability tests

Deionized water droplets with and without food coloring and the DSA100E contact angle goniometer were used to characterize the wettability of bag surfaces. WCJBs and CJBs were also exposed to simulated rainfall for ~ 1 min: they were placed at an angle of ~ 30° and the simulated raindrops were applied by releasing water droplets (~ 2 mL) from a syringe placed ~ 5 cm above the bags. The moisture absorption of WCJBs and CJBs was estimated by measuring their mass.

### Seed storage experiments

Clean and healthy wheat (*T. aestivum*) grains, devoid of any physical damage, were selected and placed inside paper bags for initial weight measurement. These grains were then dried in an oven at 80 °C for 72 h, after which their mass did not change further; thus, SMC in the initial state was calculated as follows: $SMC=({W}_{1}-{W}_{2})/{W}_{1}$, where ${W}_{1}$ is the initial weight of grains and ${W}_{2}$ is their final weight after drying. Subsequently, wheat grains were packaged in WCJBs and CJBs, and the bags were placed in airtight plastic containers with the following RHs: 55%, 75%, and 98%. The air humidity level was controlled in confined spaces by equilibrating it with supersaturated salt solutions of Ca(NO_3_)_2_·4H_2_O, NaCl, and K_2_SO_4_^80,81^. Specifically, to obtain 55% RH, 50 g of Ca(NO_3_)_2_·4H_2_O was dissolved in 100 mL of deionized water by stirring; more salt was added until a saturated solution was formed with a salt:water ratio of 3:1 (w/w). This procedure was repeated to obtain 75% RH using NaCl (but with a 2:1 salt:water ratio) and 98% RH using K_2_SO_4_ (at a ~ 3:1 salt:water ratio). The saturated salt solutions were then poured into the lower compartment of the airtight plastic containers, which were integrated with humidity and temperature sensors (Fig. S1). The bags containing wheat grains were then placed on a plastic mesh tray (barrier) above the saturated salt solutions; therefore, they were exposed to air with well-controlled humidity. Experiments were carried out in triplicate; therefore, nine containers with WCJBs or CJBs were randomly placed and monitored for 2 months. Moisture absorption was determined by weighing each jute bag every 3 days. Total moisture absorbed was calculated in grams per gram of jute bag plus stored seed combined.

### Seed moisture content and post-storage germination/viability

After 2 months of seed storage, grains from each bag was thoroughly homogenized and ~ 130 g of seed sub-sample was randomly withdrawn from each bag and put into paper bags, weighed, and then oven-dried at 80 °C for 72 h to determine post-storage SMC. To evaluate the germination efficacy of post-storage seeds, 30 grains from each storage bag were germinated in Petri dishes containing Whatman filter paper soaked in deionized water. These dishes were maintained in a dark incubator and allowed to germinate at 21.6 ± 0.5 °C. The germination patterns of seeds were monitored from the first day after sowing until the seventh day, after which the experiment was discontinued.

### Statistical analysis

The seed storage experiment involved a completely randomized factorial design, which was used to investigate the effect of jute storage bags and RH on moisture absorption, SMC, and seed germination percentage. For analysis of data including triplicate samples per treatment, we used Origin Pro software to perform a two-way analysis of variance (ANOVA), with Tukey’s test applied for multiple comparisons of means; *p* < 0.05 was set as the level of statistical significance^82^.

## Supplementary Information

Supplementary Video 1.

Supplementary Video 2.

Supplementary Information 1.

## Data Availability

All data supporting the findings reported in this study are available in the paper.
