# Supplementary material for: Nature-inspired wax-coated jute bags for reducing post-harvest storage losses
Source: Sci Rep. 2021 Jul 28;11:15354. doi: 10.1038/s41598-021-93247-z (PMC8319191; doi:10.1038/s41598-021-93247-z)
Supplement: Supplementary file 3 — Supplementary Information 1. [file 41598_2021_93247_MOESM3_ESM.docx]

**Supplementary Information**

**Nature-inspired Wax-coated Jute Bags for Reducing Post-Harvest Storage Losses**

Kennedy Odokonyero, Adair Gallo Jr., and Himanshu Mishra^*^

King Abdullah University of Science and Technology (KAUST), Water Desalination and Reuse Center, Division of Biological and Environmental Sciences and Engineering,

Thuwal 23955 - 6900 Saudi Arabia,

^*^Corresponding Author: [himanshu.mishra@kaust.edu.sa](mailto:himanshu.mishra@kaust.edu.sa)


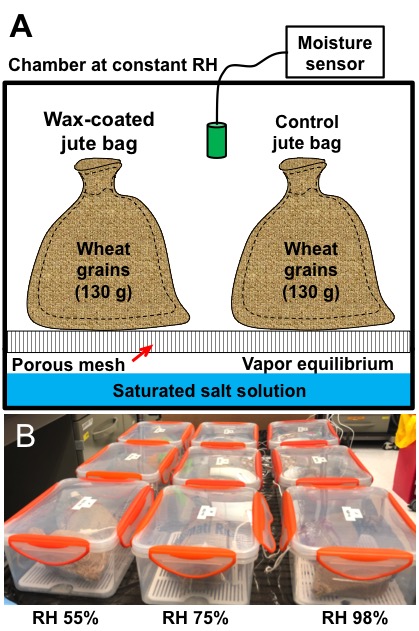


**Figure S1.** Moisture absorption setup for wheat grains stored in jute bags under variable relative humidity (RH) conditions. (**A**) Schematic of a moisture box containing one control jute bag (CJB) and one wax-coated jute bag (WCJB) in each seed storage box. Each bag contained 130 g of oven-dried wheat grains. The saturated salt solution at the bottom of the box allowed for precise control of air RH. (**B**) Photograph of the experimental setup showing the three different RH conditions (50%, 75% and 98% RH) replicated in triplicate.
